# A novel *in situ* permeation system and its utility in cancer tissue ablation

**DOI:** 10.3892/ijo.2015.3068

**Published:** 2015-06-29

**Authors:** MASAMI WATANABE

**Affiliations:** 1 Center for Innovative Clinical Medicine, Okayama University Hospital, Okayama University, Okayama 700-8558, Japan; 2 Department of Urology, Okayama University, Okayama 700-8558, Japan

**Keywords:** *in situ* permeation, perfusion, convection-enhanced delivery, cancer, local therapy

## Abstract

Focal ablation therapy is an emerging treatment modality for localized cancer lesions. It is an attractive strategy for inhibiting tumor progression and preventing morbidity associated with open surgery. As for intratissue drug delivery systems for use in local therapy, the convection-enhanced delivery (CED) of liquid drugs has been utilized, particularly for the treatment of malignant brain tumors. Although the conventional CED system is useful for providing drug/vehicle-based local therapy, there are several reported disadvantages in terms of the ability to control the extent of drug diffusion. We herein developed and validated a novel *in situ* permeation (ISP)-MW-1 system for achieving intratissue drug diffusion. The ISP system includes a perfusion catheter connected to an injector and aspirator, which enables intratissue perfusion of the solute diluted in the vehicle in the tip-inserted cavity. We subsequently evaluated the utility of the ISP-MW-1 system for *in situ* permeation in a subcutaneous tumor model in hamsters. Dehydrated ethanol, saline and 50% acetic acid were evaluated as the vehicle, and methylene blue was used as a dissolved substance for evaluating the diffusion of the agent. As a result, almost all of the tumor tissue within the capsule (tumor size: ~3 cm) was permeated with the dehydrated ethanol and 50% acetic acid and partially with the saline. We further demonstrated that ISP treatment with 50% acetic acid completely ablated the subcutaneous tumors in all of the treated hamsters (n=3). Therefore, the ISP-MW-1 system is a promising approach for controlling the intratissue diffusion of therapeutic agents and for providing local ablation therapy for cancer lesions. We believe that this system may be applicable to a broad range of medicinal and industrial fields, such as regenerative medicine, drug delivery systems, biochemistry and material technologies as well as cancer therapy.

## Introduction

In the early phase of carcinogenesis, the tumor often remains focal and restricted to the organ of origin. For the treatment of localized cancer lesions, a focal therapeutic approach with minimal morbidity is the ideal modality. Percutaneous chemical ablation is an established local therapy for small hepatocellular carcinoma, with several previous studies demonstrating comparable safety, efficacy and long-term survival to that seen with surgical resection (1–3). Chemical ablation methods have also been reported to have the advantages of being inexpensive, rapid and simple (3). Additionally, the direct local injection of anti-oncologic or tissue-ablative agents has been performed successfully to treat various types of tumors, without major complications, including malignant brain tumors (4,5), renal carcinoma (6), adrenal neoplasms (7) and prostatic hyperplasia and adenocarcinoma (8,9).

Convection-enhanced delivery (CED) is a technique that relies on the bulk flow established by a pressure gradient over time to ‘push' the infused drug away from the catheter, resulting in continuous diffusion and the widespread distribution of the infusate within the target tissue (10–14). In the CED method, the intratissue interstitial space between the cells is utilized as the pathway for drug delivery. The diffusion of the drugs usually depends on the free concentration gradient and diffusivity of the agent itself into the tissue (10). On the other hand, intratissue fluid convection and the bulk flow are dependent on the intratissue pressure gradient and are involved in the diffusion of the agent. Since the pressure-dependent bulk flow of interstitial fluid occurs following the direct intratissue infusion of medical agents, the convection can be used to enhance the diffusion of the drugs and treat a much larger volume of the target tissue than that achieved via diffusion alone (10,14).

Although the CED technique has been applied clinically as a focal approach to treat malignant brain tumors (4,5) and benign prostatic hypertrophy (8) with an appropriate agent (drug diluted in the vehicle), there are several reported disadvantages in terms of the degree of control of drug diffusion. The extent of infusate distribution achieved using the CED method is affected by many factors, including the type of tissue infused (that is, normal tissue versus tumor tissue), interstitial pressure in the extracellular space, molecular weight of the infusate, infusion volume and rate and diameter and type of infusion catheter (11,12). The catheters used for CED infusion have a lumen from which the infusate is infused, and once the infusate is released from the lumen, the infused agent flows unexpectedly to the path of the lowest interstitial pressure (11). As for clinically observed issues in brain tumor trials of CED infusion, an uneven distribution and leakage of the drug into unexpected areas and spaces have been reported (11,12). The other major problem hindering effective CED-based drug delivery is the incidence of infusate reflux or ‘backflow' along the catheter/tissue interface of the catheter track (12,14). This reflux causes the infusate to flow away from the target tissue, thus reducing the chance of achieving a therapeutic drug concentration in the target structure and increasing the risk of off-target side effects (12). Therefore, in order to overcome these disadvantages in the CED technique and make local therapy more a useful modality, it is necessary to develop a novel type of infusion device enabling the more controlled and precise diffusion of the agent.

Based on this background, we developed a novel method for achieving intratissue diffusion by permeating the infusate *in situ*, termed the *in situ* permeation (ISP) system, which is based on the principle of ISP-MW-1. The purpose of this technique is to control the extent of intratissue diffusion and achieve widespread distribution of the infusate. These aims include preventing uncontrolled diffusion and backflow along the outside of the catheter and allowing for more homogeneous drug delivery within the target tissue, thereby overcoming the disadvantages observed with the conventional CED system. For the goal of attaining intratissue drug diffusion, the ISP-MW-1 system contains a perfusion catheter connected to an injector and aspirator, which enables the intratissue perfusion of the solute diluted in the vehicle in the tip-inserted cavity. In comparison to the CED system, the ISP-MW-1 system is quite different and inventive because the various perfusion-related parameters are controllable by changing the infusion speed and aspiration pressure. In addition, the aspiration-based vacuum of the ISP-MW-1 device enables the removal of the target tissue-intrinsic fluid mixed in the infused agent, which may help to reduce the intratissue pressure and enhance the extent of agent diffusion within the target tissue.

We herein evaluated and validated this novel *in situ* permeation system and device in terms of the ability to obtain local intratissue drug delivery by analyzing the degree of intratissue diffusion of liquid agents. In order to further assess the possible clinical application of the ISP-MW-1 system, we evaluated the therapeutic utility and feasibility of this technique for performing chemical ablation of cancer tissue in a subcutaneous tumor model in hamsters.

## Materials and methods

### Construction of the in situ permeation (ISP)-MW-1 system

**Figure 1:**
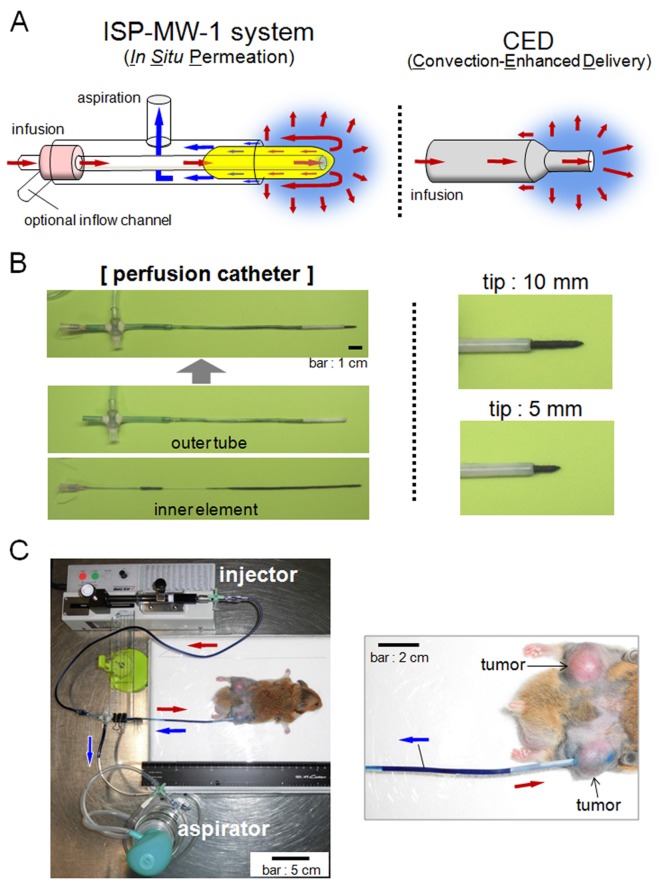
(A) Schematic figure showing the principle of the ISP-MW-1 system and conventional CED system-based intratissue drug delivery. Both systems are used to achieve the local diffusion and distribution of medical agents. The flow of the infused agent is indicated by red or blue arrows. In the ISP-MW-1 system, another inflow channel is optionally added for the air inflow (explained in Fig. 3C) or infusion of a second agent. (B) The image of the perfusion catheter used in the ISP-MW-1 system. The inner element is inserted into and invaginated to the outer tube (left panel). The tip of the inner element is covered with cotton, and the cotton cover is proximally prolonged into the outer aspiration tube, through which the infused agent can be efficiently absorbed and smoothly moved into the outer tube spontaneously or via aspiration. The tip of the perfusion catheter is scalable in the length for use based on the size of the target tumor or tissue (right panel). (C) The entire picture of the device during the ISP-MW-1 procedure for the treatment of subcutaneous tumors with dehydrated ethanol (methylene blue was dissolved as a dye) in the hamster model (left panel). In the ISP-MW-1 system, a perfusion catheter is connected to tubes attached to an injector and hand-operated aspirator, which enable the intratissue perfusion of the agents in the tip-inserted cavity. The injector consists of the syringe and microsyringe pump, and the flow rate of infusate is controlled with the micropump. The flow of the agent is indicated by red (infusion) or blue arrows (spontaneous flow or aspirated flow). An enlarged view is shown in the right panel.

The principle of the ISP-MW-1 system-based intratissue drug delivery is shown in Fig. 1A, the system used herein to achieve the local diffusion and distribution of the liquid agents. In the ISP-MW-1 system, another inflow channel is optionally added for the air inflow (explained in Fig. 3C) or infusion of a second agent. The perfusion catheter is inserted and placed in the target tissue and used for *in situ* permeation. The catheter consists of an outer tube and inner element, the latter of which is inserted into and invaginated to the outer tube (Fig. 1B). The working tip of the perfusion catheter is scalable in length, so that the tip at the cavity can be adjusted to the preferable length based on the size of target tumor or tissue. Fig. 1B shows the appearance of the tip at the 10- and 5-mm lengths. The thickness of the tip of the perfusion catheter was approximately 1.5 mm in this study. The tip of the inner element is covered with cotton, and the cotton cover is proximally prolonged into the outer aspiration tube, through which the infused agent can be efficiently absorbed and smoothly moved into the outer tube spontaneously or via aspiration. In addition, the placement of the prolonged cotton cover in the outer aspiration tube prevents the entry of blood and separated small tissues into the tube and clogging during aspiration.

In the ISP-MW-1 system, a perfusion catheter is connected to tubes attached to an injector and hand-operated aspirator (Muranaka Medical Instruments Co., Osaka, Japan), which enable the intratissue perfusion of the agents in the tip-inserted cavity (Fig. 1C). The injector consists of a syringe (1005TLL, 5 ml SYR; Hamilton Co., Reno, NV, USA) and microsyringe pump (MSPE-1; AS One Co., Osaka, Japan), and the flow rate of the infusate is controlled with the micropump. The aspirator supplies a vacuum pressure of more than 150 mmHg by compressing the bulb made of silicone. We herein used an outer tube with a polypropylene component (outer diameter: 2.6 mm, inner diameter: 2.2 mm, length: 3.5 cm) at the distal head. The proximal end of the outer tube is attached to the inner element tubing to the infusion micropump. The aspiration tube of the outer tube is directly connected to the hand-operated aspirator. In order to adjust the location of the perfusion catheter, a stand that allows the catheter to be held and moved is utilized.

### Subcutaneous tumor model in hamsters

Male Syrian hamsters were used for the animal experiments in this study. The experiments employing the tumor model in hamsters were approved by the Animal Care and Use Committee, Okayama University. The animals were kept in a specific pathogen-free housing facility at Okayama University. A hamster pancreatic cancer cell line (HaP-T1) was provided by the RIKEN BioResource Center (Ibaraki, Japan) through the National Bio-Resource Project of the MEXT in Japan, and the cells were cultivated as previously described (15). The HaP-T1 cells were inoculated into the left and right femurs, and a hamster model bearing bilateral subcutaneous tumors was developed.

### Animal experiments of infusate diffusion with the ISP-MW-1 system

Prior to inserting the perfusion catheter into the tumor tissue, the animals were deeply anesthetized with pentobarbital solution via intraperitoneal injection. A small skin incision was made in the appropriate position for concentric diffusion of the agent, and a needle was placed along the direction of insertion. The tract was dilated using larger sheaths up to the outer diameter, so that the perfusion catheter may be inserted into the tract. Depending on the tumor size, either a 10- or 5-mm-long tip of the perfusion catheter was selected for *in situ* permeation in this study (Fig. 1B). The perfusion catheter was carefully inserted into the tract so that the tip was located as centrally as possible within the tumor tissue. Dehydrated ethanol, saline and 50% acetic acid (diluted with H_2_O) were evaluated as the vehicle, and methylene blue (Wako Pure Chemical Industries, Osaka, Japan) was added to the vehicle as a dye at a concentration of 2 mg/ml. Before applying the agents, the insoluble material was removed as precipitation. We used the dye to evaluate the degree of intratissue diffusion and distribution of the agent achieved with the ISP-MW-1 system, and the dye diffused edge was interpreted as indicating the extent of permeation of the agent in the *in vivo* experiments. The agent was drawn into a Hamilton syringe and the attached tube, so that no dead space was apparent. The tube was subsequently connected to the perfusion catheter, and the catheter was then flushed until the solution dripped from the tip.

After inserting the perfusion catheter into the tumor, the microsyringe pump was placed and the intratissue perfusion of the agent was started at the indicated flow rate for each treatment. The total time of ISP-MW-1 therapy was also indicated for each treatment. During the ISP procedure, periodical aspiration using the hand-operated aspirator was performed in order to vacuum the cavity and keep it at negative pressure. As explained in Fig. 3C, the aspiration-enhanced procedure was further added to remove the agent and tissue-intrinsic fluid efficiently from and around the tip cavity. Aspiration was conducted at a frequency required to avoid leakage of the infusate from the point of catheter insertion. An image of the entire device during the ISP-MW-1 procedure with dehydrated ethanol in the hamster model bearing a subcutaneous tumor is shown in Fig. 1C. When the perfusion of the infusate was complete, the perfusion catheter was slowly withdrawn over a period of 10 sec and the tumors were resected. The tumor was cut into 8- to 10-mm-thick sections, allowing for observation of the distribution of the dye within the tumor.

### Animal experiments for cancer chemical ablation using the ISP-MW-1 system

Three hamsters bearing bilateral subcutaneous tumors were used for the cancer ablation study. Before treatment, the size of all tumors was measured and the tumor volume was calculated using the previously described formula (16). On day 0, the side of treatment was randomly selected, and the tumor was treated with 50% acetic acid using the ISP-MW-1 system. Sham treatment was performed on the untreated tumor side simply by inserting another perfusion catheter. All hamsters were examined to determine the bilateral tumor volume on day 7 after treatment and then sacrificed.

### Histological procedure

For the histological observation of the HaP-T1-derived tumors, the subcutaneous tumors were dissected at the time of sacrifice of the animals. The tumor tissue was fixed in formalin and embedded in paraffin to obtain sections. The sections (5 μm) were stained with hematoxylin and eosin and photographed for histopathology.

## Results

### Whole intratissue diffusion of the chemical agents was achieved with the ISP-MW-1 system

**Figure 2:**
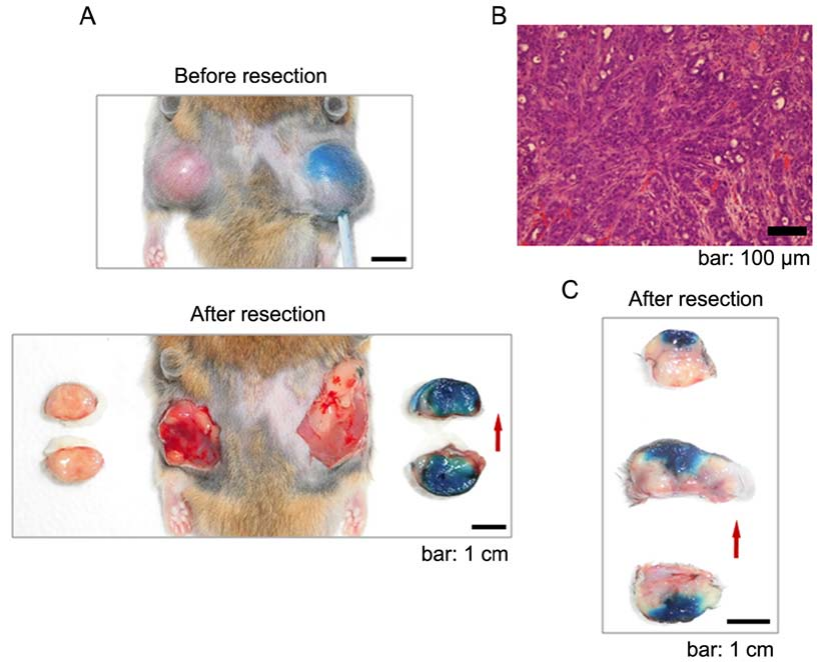
(A) The image of the subcutaneous tumors in the same hamster as in Fig. 1C immediately prior to (upper panel) and after (lower panel) resection. A total 60 min of perfusion with the ISP-MW-1 system was performed, and then both tumors were resected. Images of tumor sections indicating the full distribution of the agent are shown in the lower panel. The red arrow indicates the direction of insertion of the perfusion catheter. (B) Histological appearance of the untreated subcutaneous tumor. The sections were stained with hematoxylin and eosin. (C) The subcutaneous tumor was treated with saline (methylene blue was dissolved as a dye) for 3 h with the ISP-MW-1 system and then resected. Images of tumor sections indicating partial diffusion of the agent are shown. The red arrow indicates the direction of insertion of the perfusion catheter.

**Figure 3:**
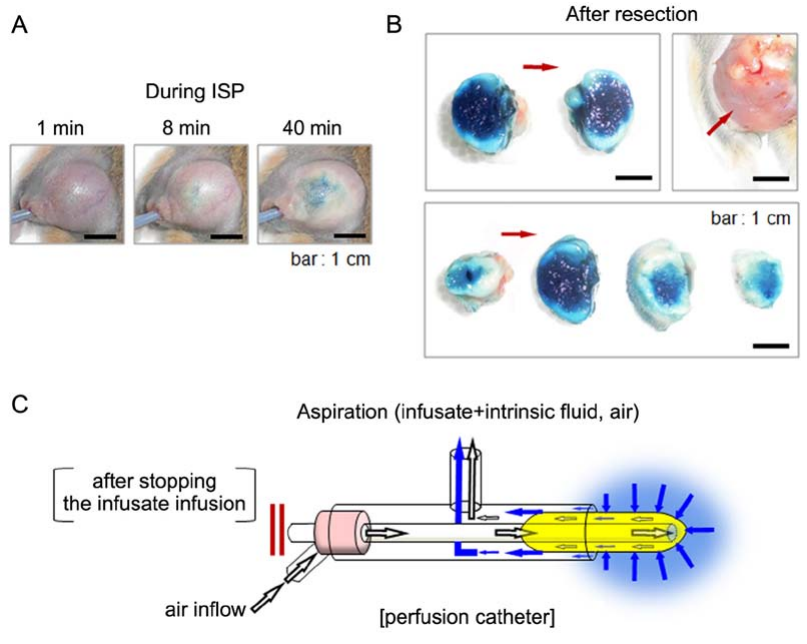
(A) The image of the subcutaneous tumor during treatment with 50% acetic acid (methylene blue was dissolved as a dye) using the ISP-MW-1 system. Before taking each photograph at 8 and 40 min of ISP treatment, the infusate and intrinsic fluid were intermittently removed via the aspiration-enhanced procedure explained in (C). The tumor size is almost the same in the three images, indicating that swelling of the tumor tissue induced by diffusion of the agent was avoided with the aspiration-enhanced procedure. (B) The subcutaneous tumor in (A) was further treated with 50% acetic acid for a total 60 min perfusion, and then the tumor was resected. Images of tumor sections in two pieces (upper left panel) and four pieces (lower panel) are shown. In both panels, full diffusion of the agent was observed. The appearance of the subcutaneous area after resection is shown in the upper right panel. The red arrow indicates the direction of insertion of the perfusion catheter. (C) Schematic figure of the aspiration-enhanced phase in the ISP-MW-1 system. For removal of the infusate and tissue-intrinsic fluid from the tip of perfusion catheter and intratissue cavity, the infusion of the infusate is temporarily stopped and another inflow channel is opened for the air inflow. In this phase, the aspiration induces a continuous air flow and circulation through the tip-covered cotton, and most of the infusate and intrinsic fluid around the tip can be efficiently aspirated to the outer tube. The procedure with the air inflow enables the prompt removal of the agent and intrinsic fluid from and around the cavity. After this phase, the status of tissue swelling and high intratissue pressure is improved, after which flesh agents are resupplied and refilled in the cavity, resulting in more efficient *in situ* permeation in the target tissue.

In order to investigate the utility of the ISP-MW-1 system for achieving the *in vivo* focal distribution of a liquid drug, a subcutaneous tumor model in hamsters was used. The histopathology of the tumors indicated solid and malignant proliferation of cancer cells (Fig. 2B). Dehydrated ethanol, saline and 50% acetic acid were used as the vehicle, and the findings of intratissue diffusion are shown in Figs. 2A and C, and 3B, respectively. The perfusion parameters of the perfusion catheter and ISP-MW-1 treatment were as follows: [dehydrated ethanol; tip length: 10 mm, flow rate: 50 μl/min, total perfusion time: 60 min], [saline; tip length: 10 mm, flow rate: 100 μl/min, total perfusion time: 3 h] and [50% acetic acid; tip length: 10 mm, flow rate: 100 μl/min (first 45 min) and 250 μl/min (latter 15 mins, total perfusion time: 60 min]. Within 10 min of perfusion with the ISP-MW-1 system, noticeable dye staining was typically observed through the tumor skin (Figs. 1C and 3A). Regarding the results for the diffusion experiments with dehydrated ethanol and 50% acetic acid, the agent was distributed throughout the tumor tissue in each section and confined within the area of the tumor, with no significant leakage into the peritumoral space (Figs. 2A and 3B). When saline was used as the vehicle, the intratissue diffusion into the tumor tissue was relatively partial in comparison to that observed with the other two vehicles (Fig. 2C). When 50% acetic acid used as the vehicle, the edge of intratissue diffusion usually presented as a white band (Fig. 3B), indicating that only acetic acid had reached that area. In the ISP-MW-1 system, the flow rate (injection speed) can be controlled with the microsyringe pump, which may have influenced the extent of agent diffusion into the tumor tissue. No obvious leakage of the infused agents was observed from the catheter/tissue interface of the point of insertion during the ISP-MW-1 procedure. In addition, upon withdrawal of the perfusion catheter after the completion of perfusion, no leakage of the agents was observed at the point of insertion of the perfusion catheter. No apparent side effects were observed during the ISP-MW-1 procedure in the hamsters treated with the three types of vehicle.

### An aspiration-enhanced phase was added as a step to the ISP-MW-1 system

In order to control the rate of diffusion and distribute a greater volume of agent, we herein attempted to remove the infused agent and target tissue-intrinsic fluid surrounding the tip of the catheter. As shown in Fig. 3C, an aspiration-enhanced procedure was intermittently added as a step to the ISP-MW-1 system in the experiments conducted with 50% acetic acid as the vehicle. In order to obtain the prompt and efficient removal of the fluid from and around the tip cavity, the air inflow was induced to the tip and the air was subsequently aspirated with the agent and intrinsic fluid through the outer tube. In the aspiration-enhanced phase, the air was taken through the optional inflow channel after stopping the infusate infusion (Fig. 3C). Consequently, the tumor size remained almost the same during the ISP-MW-1 procedure (Fig. 3A), indicating that swelling of the tumor tissue as a result of diffusion of the agent was avoided with the aspiration-enhanced procedure. These results also suggest that both the infusate and intrinsic fluid at or around the tip were smoothly eliminated in the ISP-MW-1 step, which increased the intratissue standby capacity and enhanced the consequent agent flow and diffusion within the tissue.

### Complete tumor ablation was achieved with the perfusion of 50% acetic acid using the ISP-MW-1 system

**Figure 4:**
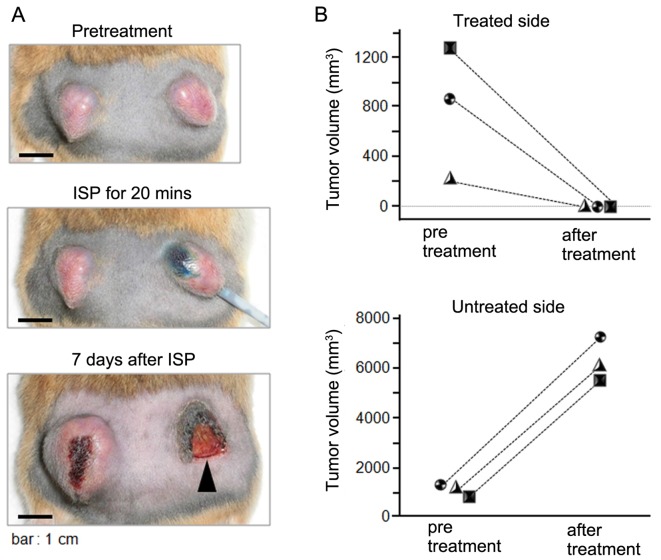
(A) The utility of the ISP-MW-1 system for achieving cancer ablation was evaluated in the bilateral subcutaneous tumor model in hamsters. The right tumor was treated with 50% acetic acid (methylene blue was dissolved as a dye) using the ISP-MW-1 system. In the left tumor, the perfusion catheter was temporarily inserted as a sham operation. The images of the subcutaneous tumors before treatment, at 20 min of ISP treatment and on day 7 after ISP treatment are shown. A total 20 min of perfusion of the agent was performed in the right tumor. The arrowhead indicates the scar without any residual cancer tissue. (B) The antitumor effects of the local therapy were evaluated using three tumor-bearing hamsters. An image of the treatment course in one of the hamsters is shown in (A). The length of the tip of the perfusion catheter and total time for perfusion with acetic acid using the ISP-MW-1 system were altered in reference to the tumor size prior to treatment. All hamsters were examined for the tumor size on day 7 after ISP with the agent. The untreated tumors were also monitored as a negative control. The graph indicates the tumor volume on the treated and untreated sides. On the treated side, all tumors completely disappeared following chemical ablation with ISP-MW-1 (upper panel). In contrast, the tumor volume rapidly increased on the untreated side (lower panel).

Based on the promising results obtained in the dye diffusion studies, we next explored the utility of the ISP-MW-1 system for achieving therapeutic tumor ablation. The anti-tumor effects of the local therapy with 50% acetic acid were evaluated using three tumor-bearing hamsters (Fig. 4). In the tumors treated with acetic acid, the length of the tip of the perfusion catheter and the total time for perfusion was altered in reference to the tumor size prior to treatment. The perfusion parameters of the perfusion catheter and ISP-MW-1 treatment (Fig. 4B) were as follows: [hamster 1 (square symbol); tip length: 10 mm, flow rate: 100 μl/minute, total perfusion time: 20 min], [hamster 2 (circle symbol); tip length: 10 mm, flow rate: 100 μl/min, total perfusion time: 10 min] and [hamster 3 (triangle symbol); tip length: 5 mm, flow rate: 100 μl/min, total perfusion time: 5 min]. In the untreated tumors, the perfusion catheter was temporarily inserted as a sham operation, and the tumor growth was monitored as a negative control. As shown in Fig. 4B, all treated tumors completely disappeared following chemical ablation on day 7 after the treatment. An image of the treatment course in one of the hamsters is shown in Fig. 4A and only scar, without residual cancer tissue, was observed on the treated side. On the other hand, the tumor on the untreated side had rapidly grown. No apparent complications were observed in the hamsters during the cancer ablative therapy. Therefore, significant therapeutic effects were obtained using the chemical ablation method with the ISP-MW-1 system.

## Discussion

We evaluated the utility of the ISP-MW-1 system for achieving the *in situ* permeation of liquid agents in a subcutaneous tumor model in hamsters. Dehydrated ethanol, saline and 50% acetic acid were employed as the vehicle, and methylene blue was used as a dissolved substance to assess the extent of agent diffusion. As a result, almost all of the tumor tissue within the capsule (tumor size: ~3 cm) was permeated with dehydrated ethanol and 50% acetic acid and partially with saline. We further demonstrated that ISP treatment with 50% acetic acid completely ablated the subcutaneous tumors in all of the treated hamsters. Therefore, the ISP-MW-1 system is a promising approach for achieving an optimal intratissue drug/vehicle distribution and providing local ablation therapy for cancer lesions.

**Figure 5:**
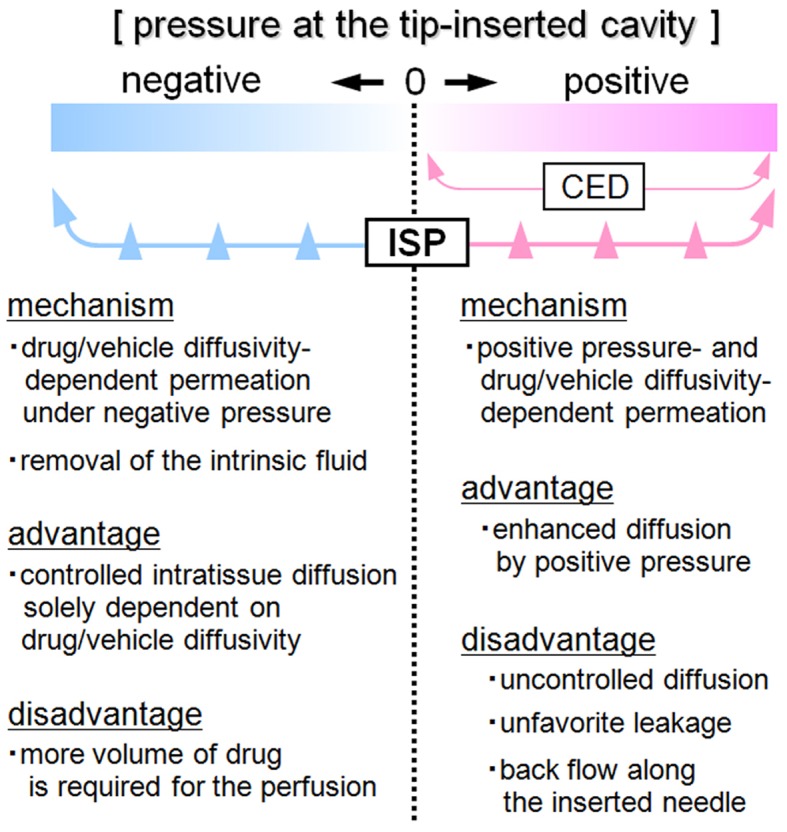
Using the ISP-MW-1 system, the intratissue pressure in the tip-inserted cavity can be changed between positive and negative. In the positive pressure mode, the mechanism of *in situ* permeation is similar to that observed in the CED system. Under the perfusion with ISP-MW-1 system, the degree of intratissue diffusion is dependent on the convection due to the effects of the positive pressure and the drug/vehicle diffusivity. When keeping the cavity pressure periodically negative, the focal diffusion of the infused agents is more controllable. Additionally, when maintaining negative pressure with the ISP-MW-1 system, the tissue-intrinsic fluid can be aspirated and removed with the infused agent through the tissue-catheter tip interface.

To our knowledge, ISP-MW-1 is the first infusion device that enables the clinician to control the extent of *in situ* permeation by changing the pressure in the tip-inserted cavity from positive to negative (Fig. 5). When the cavity pressure is maintained positive by setting the appropriate infusion speed and aspiration pressure, the ISP-MW-1 works as a CED system and can be employed to acquire more enhanced diffusion in comparison to that seen with negative pressure. On the other hand, when the pressure is maintained to be negative, more controllable diffusion is expected due to the diffusivity of the agent itself. In this study, we validated the ability of the ISP-MW-1 system and its perfusion catheter for achieving controllable *in situ* perfusion of the liquid agents. Intratissue perfusion was performed under conditions of controlled infusion and aspiration, and there were no apparent signs of leakage of the infusate from the point of catheter insertion. The tip of the inner element is covered with cotton and the cotton cover is prolonged into the outer aspiration tube, through which the liquid agent smoothly moves into the outer tube for aspiration. In addition, the placement of the prolonged cotton cover in the outer aspiration tube prevents the entry of blood and separated small tissues into the tube and clogging during aspiration. It was important that the pressure in the tip-inserted cavity be periodically kept negative in all experiments in this study. The type of aspirator used in the current study was useful for maintaining a negative cavity pressure, as confirmed in additional experiments (data not shown). Due to the effects of aspiration, intratissue perfusion can be promoted through the distal to proximal part of the tip of the perfusion catheter. In addition, perfusion of the solute and vehicle continuously supplies fresh agents to the target tissue, which subsequently promotes the concentration-dependent intratissue diffusion of the agent. When the cavity pressure is kept negative using the ISP-MW-1 system and there is no positive pressure-dependent fluid convection, the diffusion depends solely on the drug permeation induced by the concentration gradient and diffusivity of the agent in the target tissue.

One obstacle to achieving optimal intratissue drug diffusion is the presence of limited interstitial space and/or low capacity of the target tissue to accept the fluid flow. In order to overcome these issues, we developed the ISP-MW-1 system with the goal of removing the tissue-intrinsic fluid and widening the interstitial pathway. In the perfusion phase of the ISP-MW-1 system, the intrinsic fluid mixed with the infusate can be aspirated and removed continuously. Furthermore, in the aspiration-enhanced phase with the air inflow after stopping the infusate infusion, both the infusate and intrinsic fluid at or around the tip may be efficiently eliminated and replaced with air (Fig. 3C). For this purpose, the cotton-covered tip is important for absorbing the fluid in the cavity and aspirating this fluid out to the outer tube. Hence, using the ISP-MW-1 device, we established a procedure for achieving intratissue fluid removal in order to increase the standby capacity and thus enhance the agent flow and degree of diffusion within the tissue. It is likely that tumor swelling and intratissue pressure elevation due to the diffusion of the agent may be efficiently improved using the aspiration-enhanced procedure, as the tumor size remained almost same during the ISP-MW-1 procedure in this study (Fig. 3A).

It is notable that the diffusion of acetic acid obtained with the ISP-MW-1 system reached the whole tumor and was limited to within the tumor tissue. The advantages of controlled intratissue diffusion have also been demonstrated in experiments with ethanol. We consider that infusion with the ISP-MW-1 system achieves a homogeneous distribution of infusate and improves the extent of controllability of *in situ* permeation via the following mechanisms (Fig. 5): i) performing focal perfusion under negative cavity pressure enables the diffusion to solely depend on the drug permeation induced by the concentration gradient and diffusivity of the agent into the target tissue, ii) removing the intratissue fluid, including the tissue-intrinsic fluid, increases the standby capacity of the extracellular space and makes it easy to control the subsequent agent permeation within the tissue. As for other advantages of the ISP-MW-1 system, the tip of the perfusion catheter is scalable in both length and thickness. The tip length and thickness should be altered for proper use based on the size of the target tumor or tissue. In addition, the outer diameter (OD) of the outer tube of the perfusion catheter can be also altered based on the target situation in each case. We used 2.6-mm OD outer tubes in the present study and are currently developing thinner tubes with an OD of ~1.2 mm, thereby enabling more non-invasive access to the target tissue. Preliminary tests of the thinner perfusion catheter are already underway to assess the utility for intratissue diffusion. On the other hand, it should be noted that there are disadvantages with respect to *in situ* permeation with the ISP-MW-1 system. Under negative cavity pressure, it is conceivable that the degree of intratissue permeation may be limited due to the lack of promoting power. Since the ISP-MW-1 procedure requires maintenance of the perfusion of the infused drug, a greater volume or amount of the drug is consumed in comparison with the CED system.

The application of ISP-MW-1 treatment with 50% acetic acid eradicated the hamster tumors in this study, consistent with the findings that whole intratissue diffusion of the tumor can be successfully achieved using the ISP-MW-1 procedure. Many studies have demonstrated the effectiveness of chemical tumor ablation using a variety of chemical liquid agents-ethanol, carbolic acid, acetic acid and glycerin (2,6–8,17). Among these agents, acetic acid has been reported to be a very strong ablative agent and is utilized in the clinical field (2,7,18). However, has also been reported that the direct injection of acetic acid into hepatomas in a rat model results in a significantly high rate of death, likely due to uncontrolled leakage of the agent into other spaces (19). Since the chemical ablative agents used in local therapy are usually cytotoxic, the ability to control the extent of intratissue diffusion and distribution is essential for preventing off-target side effects. In this study, we demonstrated that the ISP-MW-1 system is a promising approach for obtaining controlled intratissue diffusion of therapeutic agents and providing local ablation therapy for cancer lesions.

To date, a variety of local therapies using drugs have been developed for the treatment of cancer and non-cancer diseases (20–25). We expect that the ISP-MW-1 system may be used to enhance the effectiveness of local therapies by achieving widespread intratissue diffusion of these drugs. It is also expected that the improved degree of controllability of intratissue drug delivery will expand the application of local therapies for various diseases and promote the development of novel drugs and vehicles for use in local therapy. In the protocol for local therapy, the choice of vehicle for the drugs is particularly important for each individual disease. As for cancer therapeutics, cytotoxic chemical agents are available as a vehicle. If commonly used chemotherapeutic drugs could be dissolved as solutes in chemical vehicles, the compound agents would exert much greater ablative effects for the target tumor. On the other hand, regarding treatment for local lesions of non-cancer diseases, gene therapeutic approaches, cellular transplantation and anti-pathogenic drugs are available to locally reconstruct the tissue functions and eliminate the pathogens. In any case, the vehicle for the drug should not impede the intrinsic efficacy of the drug itself. In order to utilize the ISP-MW-1 system in the clinical setting, feasibility studies are needed, taking into consideration the characteristics of each disease and the investigated drug.

We herein demonstrated that the *in situ* permeation method using the ISP-MW-1 system is useful for controlling and increasing the intratissue distribution of locally infused agents. Regarding the possible clinical application of ISP-MW-1, intra-operative imaging guidance with computed tomography (CT), ultrasonography or magnetic resonance imaging (MRI) may help to yield a more accurate distribution of the infused agents. We believe that this system enables improved efficacious infusion of therapeutic agents and further elicits the effectiveness of local therapies as a minimally invasive option. We finally note that the ISP-MW-1 method may be applicable to a broad range of medicinal and industrial fields, such as regenerative medicine, drug delivery systems, biochemistry and materials technologies, as well as cancer therapeutics.
